# Change in hydration indices associated with an increase in total water intake of more than 0.5 L/day, sustained over 4 weeks, in healthy young men with initial total water intake below 2 L/day

**DOI:** 10.14814/phy2.13356

**Published:** 2017-11-17

**Authors:** Jodi D. Stookey, Janice Hamer, David W. Killilea

**Affiliations:** ^1^ Children's Hospital Oakland Research Institute Oakland California

**Keywords:** Biomarker, Healthy adults, Hydration, Water intake

## Abstract

This secondary data analysis addressed gaps in knowledge about effects of chronic water intake. Longitudinal data from the Adapt Study were used to describe effects of prescribing a sustained increase in water intake relative to baseline, for 4 weeks, on multiple indices of total body water (TBW) flux, regulation, distribution, and volume in five healthy, free‐living, young men, with mean total water intake initially below 2 L/day. Indices were measured weekly. Within‐person fixed effect models tested for significant changes in indices over time and associations between changes in indices. Agreement between indices was described. Mixed models tested if baseline between‐person differences in hydration indices modified changes in indices over time. Body water flux: The half‐life of water in the body decreased significantly. Body water regulation: Serum osmolality decreased significantly. Urine anti‐diuretic hormone, sodium, potassium, and osmolality decreased significantly. Plasma aldosterone and serum sodium increased significantly. Body water distribution: No significant changes were observed. Body water volume: Saliva osmolality decreased significantly. Body weight increased significantly by a mean ± SEM of 1.8% ± 0.5% from baseline over 4 weeks. Changes in indices were significantly inter‐correlated. Agreement between indices changed over 4 weeks. Baseline saliva osmolality significantly modified responses to chronic water intake. The results motivate hypotheses for future studies: Chronic TBW deficit occurs in healthy individuals under daily life conditions and increases chronic disease risk; Sustained higher water intake restores TBW through gradual isotonic retention of potassium and/or sodium; Saliva osmolality is a sensitive and specific index of chronic hydration status.

## Introduction

The Institute of Medicine set Adequate Intake (AI) recommendations for drinking water to prevent adverse acute effects of dehydration (IOM, 2004). The recommendations are, explicitly, not designed to lower risk related to chronic effects of dehydration (IOM, 2004, p4): “Although a low intake of total water has been associated with some chronic diseases, this evidence is insufficient to establish water intake recommendations as a means of reducing the risk of chronic disease. Instead an AI for total water is set to prevent deleterious (primarily acute) effects of dehydration, which include metabolic and functional abnormalities.” Controversy over the concept of chronic dehydration (Cheuvront and Kenefick 2016) and gaps in knowledge about the effects of chronic or repeated intake of specific amounts of water (IOM, 2004; Armstrong 2012; El‐Sharkawy et al. 2015; Cheuvront and Kenefick 2016) restrict the scope of the AI recommendations.

The concept of chronic dehydration is controversial in the scientific literature, because chronic total body water deficit is generally believed to *not occur* in healthy, free‐living individuals, with ad libitum access to food and beverages (Weitzman and Kleeman 1979; IOM, 2004; Cheuvront and Kenefick 2016). Under resting, temperate conditions, healthy adult men reportedly regulate water balance to within ±0.2 percent of body weight (Adolph 1943). Although fluid intake may not match fluid loss over short periods, “over an extended period, fluid consumption will match body water needs (if adequate amounts are available) (IOM, 2004, p106)”. Water turnover studies assume that the water intake of non‐institutionalized healthy adults is in balance with water efflux (Raman et al. 2004). In nationally representative data, the mean serum osmolality, “the primary physiological signal used to regulate water balance,” is stable across a wide range of total water intakes (IOM, 2004, p112‐115).

Consensus about stable total body water in healthy individuals is based on studies involving *ad libitum* water intake and change in body weight monitored *over short periods* of time (e.g., <1 week) (Adolph 1947; IOM, 2004). Supporting population‐based data analyses are *cross‐sectional,* and check for variation in the *mean value* of *only one hydration index*, serum osmolality, across the range of total water intake (IOM, 2004). With these study conditions and methods, chronic total body water deficit has not been detected.

It remains to be confirmed that prescribed increases or decreases in water intake, sustained over periods longer than a few days (e.g., months), do not alter hydration indices or total body water. Ad libitum water intake is subject to unconscious, involuntary dehydration, where individuals drink to satiety but a water deficit remains (Nose et al. 1988a,b; Greenleaf 1992). Between‐person differences in ad libitum water intake, which may be confounded by between‐person differences in water requirement, are not the same as within‐person changes in water intake of specified volume. Effects of prescribed volumes of water intake may more easily translate into water intake recommendations than ad libitum intake.

Longitudinal studies of long‐term effects of water intake are needed to confirm cross‐sectional observations. Cross‐sectional studies cannot tease apart endogenous effects of water intake on hydration indices. While lower water intake may cause higher serum osmolality; higher serum osmolality, via thirst, might also cause higher water intake. Longer‐term adaptive responses to chronic osmotic stress are, furthermore, distinct from short‐term compensatory responses to osmotic stress (Yancey et al. 1982).

Analyses are needed to check for effects of water intake on multiple hydration indices, accounting for potential confounding variables and effect modification. Analysis of mean values, only, ignores potential for a range of biomarker response, despite recognition of “extreme variability in water needs” (IOM, 2004, p 24). Individual hydration indices are not sensitive to all types of body water loss (hypo‐, iso‐, or hypertonic), and may interact with or depend on other factors that regulate body water, such as osmoreceptor thresholds or set‐points (Mange et al. 1997; Armstrong 2007; Cheuvront et al. 2013; Cheuvront and Kenefick 2016). Serum osmolality is sensitive to water deprivation, if water loss exceeds solute loss, but may not reflect isotonic dehydration (IOM, 2004). At a point in time, serum osmolality may not reflect underlying processes of body water flux or regulation (Perrier et al. 2014).

This secondary data analysis used *longitudinal* data collected for the Adapt Study (Stookey et al. 2013) to describe changes in *multiple indices* of body water flux, regulation, distribution, and volume associated with *prescribed* increases in total water intake, *sustained over 4 weeks,* in healthy, free‐living, young men, with mean total water intake below 2 L/day at baseline. The analysis described group mean values, between‐person variation, and agreement between indices before and after the chronic increase in water intake. The analysis checked for interdependent change in body water flux, regulation, distribution and/or volume. The analysis provides preliminary data to inform future studies of chronic water intake.

## Methods

### Study design

The study design and protocol is described in detail elsewhere (Stookey et al. 2013). The study involved four 2 week periods in an A‐B‐C‐A within‐person design. Baseline status was assessed in Weeks 1&2. In Weeks 3&4, participants were instructed to increase the total water intake from below 2 L/day at baseline to 3 L/day by consuming an additional 1 L/day (approximately 15 mL/kg/d) drinking water. In Weeks 5 & 6, participants were instructed to further increase total water intake to 4 L/day by consuming 2 L/day (about 30 mL/kg/day) drinking water. They were supplied with bottled water in Weeks 3–6. This analysis focused on changes from baseline to Week 6.

### Study participants

Five healthy, normal weight, men, age 20–25y, with baseline 3‐day mean total water intake below 2 L/day, completed the protocol. At baseline, the participants were similar with respect to body weight, height, 3‐day mean physical activity, dietary intake, nonsmoking status, perceived stress, medication use, medical history, and laboratory indices (see Stookey et al. 2013 for further detail). The protocol was approved by the Institutional Review Board of Children's Hospital & Research Center Oakland, CA.

### Weekly protocol

Each week, participants kept 7d‐diet records, collected specimen, and attended a 2‐hr clinic visit. Each participant was given a copy of his Week 1 diet records and instructed to consume similar meals for the following 7 weeks. On the day before the weekly clinic visit, participants collected all urine after the first morning void until 11 pm in a Day urine collection container and all urine from 11 pm, overnight, in a night urine collection container. Participants were instructed to consume the same dinner on the evening before the clinic visit, and restrict all food and water intake after 11 pm on the night before the clinic visit. On waking the morning of the clinic visit, each participant collected his first morning urine in a First Morning urine collection container at home. On arrival at the clinic, fasting body weight was measured in duplicate using a calibrated clinical scale (Scale‐Tronix, Carol Stream, Illinois, USA) after participants voided and removed shoes and outer clothing. Fasting saliva was next collected. Participants rated their thirst using a visual analog scale, and then consumed a 750 mL bolus of drinking water. Once during each study period, 2H_2_O was added to the water bolus to index water turnover via the 7‐d deuterium elimination rate. Whole body bioelectrical impedance resistance and reactance, indices of total body water volume and distribution and volume, respectively (Jaffrin and Morel 2008), were measured in triplicate 30 min after the water bolus, with the participant lying down, using tetrapolar measurement on hand and foot and 50 kHz current frequency (Hydra 4200 Xitron Technologies, San Diego). Fasting resting metabolic rate (RMR) was estimated by indirect calorimetry 60 min after the water bolus. Blood pressure measurements and fasting blood samples were collected 90 min after the water bolus.

### Water intake

The weekly mean intakes of drinking water and total water intake were estimated for each participant from the 7‐day diet records using Nutrition Diet Systems (NDS‐R) software (1998–2008 Nutrition Coordinating Center, University of Minnesota, Minneapolis, MN, USA). Each week, a certified NDS diet interviewer reviewed the records for completeness, and missing details were addressed with the participant during the weekly clinic visit. The interviewer probed for beverage intake. Total water intake was calculated as the sum of drinking water, water from other beverages, water from food, and metabolic water. Metabolic water was estimated from protein, fat, and carbohydrate intake, assuming macronutrient balance and 0.41, 1.07, and 0.6 mL water/g from oxidation of protein, fat, and carbohydrate, respectively (Buskirk and Puhl 1996). Total water intake was expressed in absolute (7‐day mean L/ day) and relative terms (g/kg body weight, g/kcal energy intake, g/kcal RMR).

### Specimen collection and laboratory tests

Each week, the participants were given pre‐labeled containers, a cooler, and ice packs to collect, store, and transport the previous day and first morning urine samples. To collect unstimulated fasting saliva, participants did not swallow for 1–2 min and passively drooled through a straw into cryogenic tubes. Blood was collected in the same way each week into non‐anti‐coagulated, and heparin‐ or K_2_EDTA‐anticoagulated tubes. Manual hematocrit was determined manually in triplicate from fresh whole blood collected in 4 mL K_2_EDTA tubes. Automated hematocrit was determined from fresh whole K_2_EDTA anticoagulated blood using an Advia 120 cell counter. Whole blood was processed to separate serum from the non‐anti‐coagulated tubes and plasma from the anti‐coagulated tubes.

Urine, serum, and saliva osmolality were determined on fresh specimen, in triplicate, by freezing point depression osmometer (Advanced Instruments, Norwood, MA). Aliquots of the previous day, night, and first morning urine samples, fasting saliva, serum, and plasma were stored frozen at −80°C.

Plasma aldosterone was determined by ELISA (IBL International Corp., Toronto, Ontario, Canada). Plasma vitamin D 1,25, first morning urine sodium and potassium, and previous day 11‐h urine anti‐diuretic hormone (ADH) and norepinephrine were determined by ARUP Laboratories (Salt Lake City, Utah). The Night and First morning urine samples were not analyzed for ADH or norepinephrine (i.e. 24‐h estimates were not determined for these variables) due to budget limitations. Serum sodium and potassium were determined by Quest Diagnostics (San Jose, CA).

### Red blood cell K:Na content

Red blood cell K:Na content was studied as a proxy for Na‐K pump activity. Heparin‐anticoagulated fresh whole blood was centrifuged to remove plasma. Red cell pellets were washed in choline. RBC lysates were digested in OmniTrace 70% HNO_3_ (EMD Chemicals) overnight at 60°C with 150–200 rpm orbital shaking, then diluted to 5% HNO_3_ with OmniTrace water (EMD Chemicals). After batching, the acid lysates were clarified by centrifugation (3000*g* for 10 min) and introduced via a pneumatic concentric nebulizer using argon as the carrier gas into a Vista Pro inductively coupled plasma – atomic emission spectrometer (ICP‐AES; Varian Inc) within 1–2 h of sample preparation as described (Zyba et al., 2017). The ICP‐AES was calibrated using National Institute of Standards and Technology (NIST)‐traceable elemental standards and validated using NIST‐traceable 1577b bovine liver reference material (BLRM). Elements analyzed were Na (568.821 nm) and K (766.491 nm). The detection range for Na and K was 0.05–50 ppm and the coefficient of variations (CV) for intra‐assay and inter‐assay precision for elements measured with the NIST reference material were routinely <10%. Cs (50 ppm) was used for ionization suppression and Y (5 ppm) was used as an internal standard for all samples. All reagents and plasticware were certified or routinely tested for trace metal work. Elemental content data was summarized using native software (ICP Expert; Varian Inc) and normalized to RBC volume deteremined by CBC.

### Deuterium elimination

At baseline and once during each study period, the 750 mL water bolus administered to participants during the weekly clinic visit included 2H_2_O. The dose administered each time to all participants was 10 mL 2H_2_O (99.9 atom%, Donated from the Western Human Nutrition Research Center, UC Davis, CA). Urine samples that were collected before each dose, plasma samples collected 90 min after each dose, and urine samples collected 7 days after each dose were transported frozen to the Institut de Recherche Clinique de Montréal and GEOTOP (University of Quebec, Montreal, Canada) for determination of deuterium content by isotope ratio mass spectrometer. After oral ingestion, the 2H enrichment plateau is achieved in 90 min in serum (Jankowski et al. 2004). After 6 h, the deuterium enrichment in blood and urine are interchangeable (Lukaski and Johnson 1985; Jankowski et al. 2004). This analysis reports 7‐day 2H elimination rates following doses that increased the 90 min 2H by ≥150 ppm over the background enrichment. The 2H elimination rate was calculated using the two‐point method (Jankowski et al. 2004). The percentage change in deuterium elimination rate over time was calculated for comparison with the percentage change in reported total water intake over the same period. The deuterium elimination rate was interpreted as index of the half‐life of water in the body.

### Indirect calorimetry

Gas exchange rates were recorded at 10 sec intervals during a 5‐min measurement period with study participants lying down under a canopy mask (Quark RMR, COSMED, Chicago, USA). The recorded measurement period began approximately 5–10 min after participants quietly rested and had adjusted to the mask. Real‐time gas exchange rates were monitored, and the recorded measurement period begun when the average minute *V*O_2_ and *V*CO_2_ changed by less than 10%. The Quark RMR software averaged the 10‐sec gas exchange rates to estimate the 5‐min mean gas exchange rates and fasting RMR. Fasting RMR was evaluated as a proxy measure of Na‐K pump activity. Na‐K pump activity accounts for 19–28% of whole body ATP use (Rolfe and Brown 1997; Pirkmajer and Chibalin 2016). Change in Na‐K pump activity results in measurable changes in RMR (Lyon et al. 1995).

### Data analysis and statistics

Measures available for secondary analysis from the Adapt Study were identified. The half‐life of water in the body was available as index of body water flux. Thirst, serum osmolality, serum electrolytes, urine ADH, plasma aldosterone, urine osmolality, blood pressure, norepinephrine and vitamin D 1,25 were available indices of body water regulation processes. Vitamin D 1,25 regulates the renin‐angiotension‐aldosterone system (Li et al. 2004; Vaidya et al. 2015). Available crude indices of body fluid distribution included hematocrit, a measure of the proportion of blood volume that is occupied by cells, and height‐normalized BIA reactance, a measure that varies in proportion with body cell mass (Lukaski and Bolonchuk 1988; Scheltinga et al. 1992; Jaffrin and Morel 2008). Two measures of hematocrit were evaluated, one determined manually and the other by automated cell counter (Advia), because Advia results can be skewed by chronic hypertonicity (Stookey et al. 2007). Height‐normalized BIA resistance (Lukaski and Bolonchuk 1988; Scheltinga et al. 1992; Jaffrin and Morel 2008), saliva osmolality (Walsh et al. 2004a,b; Oliver et al. 2008; Cheuvront et al. 2010; Taylor et al. 2012; Muñoz et al. 2013; Fortes et al. 2015), and body weight were available indices of total body water (IOM, 2004).

Stata software was used for statistical analyses (Stata SE, version 9.2, StataCorp, College Station, TX). Change in hydration indices over the study period were described in terms of group means, within‐person changes, and between‐person coefficients of variation. Change was evaluated relative to the previous week and relative to baseline (i.e., 4‐week change).

The agreement between hydration indices at baseline and Week 6 were determined using the following cutoffs to define elevated serum osmolality (295 mmol/kg (Matz^1996^)), serum sodium (145 mmol/L), hematocrit (50%), and urine osmolality (800 mmol/kg (Stookey et al. 2012)). Given a normal range for saliva osmolality of 21–77 mmol/L, for healthy young adult males 5 min after rinsing with tap water (Sawinski et al. 1966), an arbitrarily higher cutoff of 100 mmol/kg was chosen to discriminate higher versus lower overnight water restricted saliva osmolality.

Within‐person fixed effect models tested for significant change from Week 1 to Week 6 in hydration indices. Fixed effect models were used to test for significant association between indices, estimate the mean change in each index of total body water associated with a unit change in each index of body water flux, regulation and distribution, and estimate the within‐person and between‐person R‐squared (percentage of variation in one variable explained by the other) for each bivariate relationship. Stratified analyses were planned for biomarkers that differentially classified participants at baseline, because change in hydration indices over time may depend on baseline hydration status. Models were stratified by baseline saliva osmolality, because saliva osmolality was the only biomarker to vary between participants at baseline. Mixed models tested for an interaction between change over time and level of baseline saliva osmolality. Scatterplots described bivariate relationships between Week 1 to Week 6 change in aldosterone, urine sodium, RBC K:Na, RMR, serum sodium, deuterium elimination rate, and body weight by baseline saliva osmolality.

## Results

### Baseline

Table 1 describes the mean total water intake of the study participants, at baseline, as well as the group mean indices of body water flux, regulation, distribution, and volume. In Week 1, the between‐person coefficients of variation for serum osmolality, serum sodium, and hematocrit were <1%, <1%, and 3%, respectively. The coefficients of variation for urine and saliva osmolality were 14% and 33%, respectively. During the baseline period, between Week 1 and Week 2, body weight changed by less than 1% for all five participants.

**Table 1:** Mean^a^ indices of total water intake and body water flux, regulation, distribution, and volume in healthy young men over 8 weeks

	Baseline		+1 L/day Drinking water^b^		+2 L/day Drinking water^b^		Return to baseline	
	Period 1		Period 2		Period 3		Period 4	
	Week 1	Week 2	Week 3	Week 4	Week 5	Week 6	Week 7	Week 8
Total water intake								
L/day^c^		2.1 ± 0.5	2.6 ± 0.4	2.6 ± 0.2	3.3 ± 0.6^k^	3.3 ± 0.4^k^^,^ l	2.5 ± 0.7	1.7 ± 0.2
mL/kcal intake		1.0 ± 0.1	1.4 ± 0.2	1.3 ± 0.1	2.2 ± 0.6^k^	1.7 ± 0.2^k^^,^ l	1.5 ± 0.5	1.0 ± 0.1
mL/kg body weight		32 ± 7	39 ± 6	39 ± 4	49 ± 8^k^	48 ± 6^k^^,^ l	37 ± 11	25 ± 3
% Change vs Week 2			21 ± 5	22 ± 8	34 ± 5^k^	37 ± 6^k^^,^ l	5 ± 12	−25 ± 21
Body water flux								
Half‐life of water change vs Week 2, %^d^				−13 ± 8		−23 ± 5^k^^,^ l		
Half‐life of water in the body, days		14.2 ± 1.2		12. 2 ± 0.9		10.8 ± 0.7^k^^,^ l		
Body water regulation								
Thirst, mm^e^	67 ± 4	59 ± 9	67 ± 6	54 ± 4	62 ± 8	64 ± 4	64 ± 5	62 ± 3
Serum osmolality, mmol/kg^f^	288 ± 1	290 ± 1	289 ± 1	287 ± 1	287 ± 1	286 ± 1^l^	288 ± 2	290 ± 1
Urine ADH, pmol/L^g^	14 ± 2	10 ± 2	9 ± 3	7 ± 2	3 ± 1^k^	7 ± 4^l^	11 ± 4	9 ± 5
Plasma aldosterone, pg/mL^f^	111 ± 17	126 ± 27	137 ± 17	145 ± 35^k^	145 ± 23	143 ± 19^l^	124 ± 27	109 ± 14
Plasma vitamin D 1,25, pg/mL^f^	41 ± 4	53 ± 7	44 ± 7	42 ± 3	47 ± 4	63 ± 6^k^, ,t	50 ± 10	54 ± 6
Serum Na, mmol/L^f^	139 ± 1	139 ± 1	139 ± 0	140 ± 1	139 ± 1	140 ± 1	141 ± 1^k^	141 ± 1
Serum K, mmol/L^f^	4.1 ± 0.1	4.2 ± 0.1	4.1 ± 0.1	4.1 ± 0.0	4.1 ± 0.1	4.1 ± 0.0	4.2 ± 0.1	4.1 ± 0.0
RBC K:Na^f^	9.5 ± 0.5	9.2 ± 0.6	9.0 ± 0.4	8.3 ± 0.6^k^	8.7 ± 0.5	8.2 ± 0.4^k^^,^ l	8.4 ± 0.4^k^	8.5 ± 0.2^k^
Fasting RMR, kcal/day^h^	2170 ± 135	2251 ± 119	2311 ± 91	2178 ± 121	2037 ± 128	2033 ± 162	2091 ± 160	1874 ± 89
Urine Na, mmol/L^i^	123 ± 15	143 ± 48	94 ± 22	107 ± 29	83 ± 23^k^	79 ± 29^k^^,^ l	109 ± 24	94 ± 7
Urine K, mmol/L^i^	66 ± 16	49 ± 15	39 ± 9^k^	38 ± 9^k^	18 ± 4^k^	30 ± 8^k^	47 ± 11	47 ± 14
Urine osmolality, mmol/kg^i^	910 ± 58	812 ± 187	706 ± 103	594 ± 79^k^	530 ± 113^k^	514 ± 124^k^^,^ l	788 ± 121	786 ± 96
Urine norepinephrine, ug/g crt^g^	28 ± 8	34 ± 4	26 ± 11	21 ± 7	31 ± 7	35 ± 13	21 ± 8	27 ± 7
Systolic blood pressure, mmHg^f^	112 ± 4	111 ± 5	113 ± 4	107 ± 3	112 ± 5	113 ± 3	113 ± 5	109 ± 4
Diastolic blood pressure, mmHg^f^	73 ± 4	68 ± 2	73 ± 4	68 ± 4^k^	70 ± 4	71 ± 4	72 ± 5	72 ± 5
Body water distribution								
BIA height‐squared/reactance, cm^2^/Ω^h^	44 ± 3	46 ± 4	44 ± 3	45 ± 3	46 ± 3^k^	45 ± 3	45 ± 3	45 ± 4
Manual hematocrit, %^f^	47 ± 1	46 ± 1	46 ± 1	45 ± 1^k^	46 ± 1	46 ± 1	45 ± 1	45 ± 1
Automated hematocrit, %^f^	45 ± 1	43 ± 1	44 ± 1	44 ± 1	43 ± 1	43 ± 1	44 ± 1	44 ± 2
Body water volume								
Saliva osmolality, mmol/kg^f^	105 ± 15	94 ± 8	102 ± 10	101 ± 10	83 ± 9^k^	86 ± 9^l^	89 ± 7	94 ± 5
BIA height‐squared/resistance, cm^2^/Ω^h^	24 ± 3	24 ± 3	24 ± 3	24 ± 3	24 ± 3	25 ± 3	24 ± 3	24 ± 3
Body weight, kg^j^	66.1 ± 1.5	66.5 ± 1.6	66.5 ± 1.6	67.1 ± 1.4^k^	67.2 ± 1.6^k^	67.3 ± 1.6^k^^,^ l	67.2 ± 1.7^k^	68.1 ± 2.1^k^
Weight change vs previous week, %		+0.5 ± 0.2	+0 ± 0.3	+1.0 ± 0.4	+0.2 ± 0.5	+0.1 ± 0.1	−0.3 ± 0.2	+0.5 ± 0.4
Weight change from Week 1, kg		+0.4 ± 0.1	+0.4 ± 0.3	+1.0 ± 0.2^k^	+1.1 ± 0.3^k^	+1.2 ± 0.3^k^^,^ l	+1.1 ± 0.4^k^	+1.3 ± 0.5^k^
Weight change vs Week 1, %		+0.5 ± 0.2	+0.6 ± 0.5	+1.5 ± 0.4^k^	+1.7 ± 0.5^k^	+1.8 ± 0.5^k^^,^ l	+1.6 ± 0.6^k^	+1.8 ± 0.7^k^

a Data are presented as mean ± SEM, *n *=* *5, except in week 8 where *n *=* *4.

b Participants were instructed to increase plain drinking water by the prescribed absolute amount, while maintaining other beverage and food intake. Actual increases in water intake are reported in the first 4 rows of the table.

c The weekly mean intake was estimated from 7 24 h records.

d The half‐life of water in the body was estimated by deuterium elimination rate in Periods 1, 2, and 3.

e Thirst and overnight, water restricted, fasting saliva osmolality was assessed on arrival at the clinic.

f Blood pressure was measured and fasting blood collected 90 min after a 750 mL water bolus.

g Urine ADH and norepinephrine were determined on urine collected after the first morning void until 11 pm on the day before the clinic visit.

h Fasting, recumbent RMR and bioimpedance reactance were determined 60 min after the 750 mL water bolus. Bioelectric impedance measures were missing for one participant due to instrument malfunction.

i Urine sodium, potassium, and osmolality were determined on first morning specimen collected at home before the clinic visit.

j Fasting body weight was measured when participants arrived at the clinic.

k Significantly different from the baseline period in fixed effect within‐person change model, *P *<* *0.05.

l Significant trend Week 1 to Week 6 (*P* < 0.05).

At baseline, all five participants had a serum osmolality below 295 mmol/kg, serum sodium below 145 mmol/L, and hematocrit under 50%. Urine osmolality was above 800 mmol/kg for all five participants. Saliva osmolality was above 100 mmol/kg for three participants, and below that value for two participants.

### Background conditions throughout the study period

As previously reported, the 7‐day mean total intake of energy, protein, carbohydrate, and sodium did not vary significantly over the study period (Stookey et al. 2013). The estimated 7‐day mean sodium intake ranged between 3249 and 4074 mg/day over Weeks 1–6, with a grand mean ± SEM of 3662 ± 199 mg/d. The mean fasting RMR and time spent doing moderate or vigorous physical activity did not vary significantly over the study period. The mean ± SEM first morning saliva cortisol was 29.0 ± 6.9 nmol/L, at baseline, and decreased significantly to 17.7 ± 4.7 over the periods of higher water intake. Room humidity and temperature, during the weekly clinic visits, did not vary significantly from 43° and 23F. Mean transepidermal water loss on the forearm did not differ significantly from the baseline value of 10 ± 1 g/m^2^/h. Regarding urine concentrating ability, after overnight food and water restriction, at baseline, the mean ± SEM urine osmolality was 910 ± 58 mmol/kg (range 819–1137 mmol/kg). Regarding urine diluting ability, within 60 min after intake of 750 mL drinking water, the mean ± SEM decrease in urine osmolality was −737 ± 58 mmol/kg (range −616 to −944 mmol/kg).

### Change in water intake

As previously reported, total water intake increased significantly between Week 1 and Week 6 (Stookey et al. 2013). Relative to body weight, mean ± SEM total water intake increased by 50%, from 32 ± 7 ml/kg to 48 ± 6 ml/kg. Relative to energy intake, total water intake increased by an average ± SEM of 0.6 ± 0.2 g/kcal.

**Figure 1:**
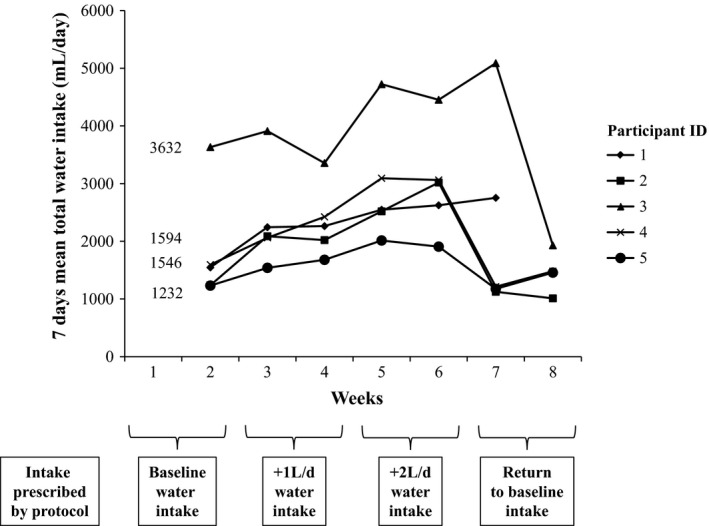
Change in total water intake for each study participant over 8 weeks.

All participants increased total water intake (see Figure 1), and maintained total water intake over 0.5 L/day higher than baseline, between Week 3 and Week 6. The magnitude of increase in total water intake relative to Week 1, that is, protocol adherence, differed by participant, ranging from +0.7 L/day to +1.7 L/day, or +32% to +51 percent in Week 6 (see Table 2).

**Table 2:** Change^a^ in indices of total water intake and body water flux, regulation, distribution, and volume in Week 6 relative to baseline for each study participant

	Change from Week 1 to Week 6 for each participant				
Participant ID	1	2	3	4	5
Total water intake					
L/day^b^	+1.0	+1.7	+0.8	+1.5	+0.7
mL/kcal intake	+0.6	+1.3	+0.6	+0.3	+0.4
mL/kg body weight	+16	+24	+11	+21	+11
% Change vs Week 2	+38	+51	+17	+46	+32
Body water flux					
Half‐life of water in the body, %^c^	−31	−38	−15	−11	−21
Half‐life of water in the body, days	−4.7	−6.7	−1.7	−1.6	−2.5
Body water regulation					
Thirst, mm^d^	−12	−4	+10	+15	−21
Serum osmolality, mmol/kg^e^	−5	−2	+1	+3	−5
Urine ADH, pmol/L^f^	+5.1	−11.8	−9.6	−16.5	+0.2
Plasma aldosterone, pg/mL^e^	+31	+15	+4	+73	+37
Plasma vitamin D 1,25, pg/mL^e^	+23	+10	+14	+27	+39
Serum Na, mmol/L^e^	−2	0	+2	+1	+3
Serum K, mmol/L^e^	−0.3	+0.3	0	+0.1	−0.1
RBC K:Na^e^	−0.7	−0.6	−1.8	−1.5	−1.7
Fasting RMR, kcal/day^g^	−5	+532	−341	−275	−595
Urine Na, mmol/L^h^	+46	−74	−81	−65	−129
Urine K, mmol/L^h^	−76	−10	−11	−10	−72
Urine osmolality, mmol/kg^h^	−342	−270	−244	−304	−817
Urine norepinephrine, ug/g crt^f^	+29	+30	+5	−28	−41
Systolic blood pressure, mmHg^e^	+2	+15	0	+5	−18
Diastolic blood pressure, mmHg^e^	−3	+1	+3	−3	−8
Serum BUN: creatinine^e^	+2	−7	−4	−1	−5
Body water distribution					
BIA height‐squared/reactance, cm^2^/Ω^g^		−0.4	+1.6	−0.3	+0.6
Manual hematocrit, %^e^	+1	−2	−1	0	−3
Automated hematocrit, %	−3	−2	−2	+2	−3
Body water volume					
Saliva osmolality, mmol/kg^d^	+10	−5	−44	−19	−36
BIA height squared/resistance, cm^2^/Ω^g^		−1	+1	+2	0
Body weight, kg^i^	+1.9	+0.6	+1.3	+1.9	+0.4
Body weight, % change	+3.0	+0.9	+2.0	+2.7	+0.6

a Data are presented as the net change from Week 1 to Week 6 for each participant, *n *=* *5, except for bioelectric impedance measures, which were missing for one participant due to instrument malfunction.

b The weekly mean intake was estimated from 7 24 h records.

c The half‐life of water in the body was estimated by deuterium elimination rate in Periods 1, 2, and 3.

d Thirst and overnight, water restricted, fasting saliva osmolality was assessed on arrival at the clinic.

e Blood pressure was measured and fasting blood collected 90 min after a 750 mL water bolus.

f Urine ADH and norepinephrine were determined on urine collected after the first morning void until 11 pm on the day before the clinic visit.

g Fasting, recumbent RMR and bioimpedance reactance were determined 60 min after the 750 mL water bolus.

h Urine sodium, potassium, and osmolality were determined on first morning specimen collected at home before the clinic visit.

i Fasting body weight was measured when participants arrived at the clinic.

### Change in body water flux

An increase in 0.5 L/day or more drinking water from baseline to Week 6 was associated with a significant mean ± SEM decrease in the half‐life of water in the body of 3.1 ± 0.8 days (*P* = 0.003) in the fixed effect, within‐person model (R‐squared within = 0.65, between = 0.67, overall = 0.15). The mean decrease in the half‐life of water in the body (−23% ± 6%) was not in proportion to the mean increase in total water intake (+37% ± 5%), however. Table 2 reports the change in half‐life of water in the body for each study participant. The response varied threefold between participants.

### Change in body water regulation processes

Serum osmolality decreased significantly from Week 1 to Week 6 (see Table 1) by a mean ± SEM of 0.5 ± 0.2 (*P* = 0.03) mmol/kg per week. Urine ADH decreased significantly from Week 1 to Week 6 by an average of 2 ± 0.5 (*P* = 0.004) pmol/L per week. Plasma aldosterone increased significantly by 7 ± 3 pg/mL per week (*P* = 0.02) during this period. Urine sodium, potassium, and osmolality decreased significantly by an average ± SEM of 11 ± 4 mmol/L, 8 ± 2 mmol/L, and 85 ± 15 mmol/kg per week, respectively. The RBC intracellular ratio of K:Na content decreased by 0.2 ± 0.08 per week (*P* = 0.004). Interim week‐to‐week changes were not statistically significant. On average, systolic blood pressure, urine norepinephrine, and serum sodium and potassium, did not vary significantly over time.

Changes over time for individual participants were heterogeneous. Serum osmolality increased, rather than decreasing, during this period for two study participants, for example (see Table 2).

### Change in body water distribution

In Week 6, the mean BIA reactance and mean hematocrit values did not differ from baseline values. No significant linear trends were observed from Week 1 to Week 6 in these measures.

### Change in body water volume

The mean height‐adjusted BIA resistance did not vary over time. Interim week‐to‐week differences in body weight were not statistically significant.

**Figure 2:**
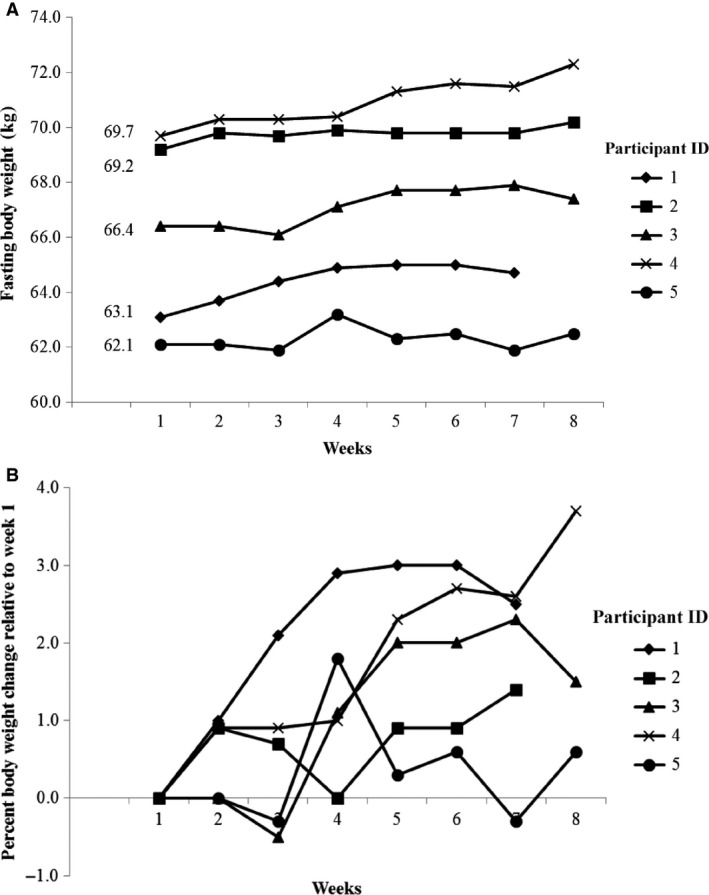
Change in body weight for each participant over the 8 week Adapt Study.

Saliva osmolality decreased significantly between Week 1 and Week 6 by an average ± SEM of 4 ± 1 mmol/kg per week (*P* = 0.009). Body weight was significantly greater relative to baseline in Week 6 (see Table 1). Body weight increased by a mean ± SEM of 1.8% ± 0.5% comparing Week 6 with Week 1. Increases in body weight were observed for all five participants, ranging from 0.6% to 3.0% (see Table 2 and Figure 2).

### Change in between‐person variation and hydration classification

After 4 weeks of sustained higher water intake, the between‐person coefficients of variation for serum osmolality, serum sodium, manual and automated hematocrit were 1%, 2%, 4%, and 6%, respectively. In Week 6, the between‐person coefficient of variation for urine osmolality was 48% versus 14% at baseline. The coefficient of variation for saliva osmolality in Week 6 was 23% versus 33% at baseline.

In Week 6, five out of five participants had serum osmolality below 295 mmol/kg, serum sodium below 145 mmol/L, hematocrit values below 50%, and urine osmolality below 800 mmol/kg. Four out of five participants also had a fasting morning saliva osmolality below 100 mmol/kg.

### Change in body water volume associated with changes in body water flux, regulation or distribution

In within‐person fixed effect models, increases in percent body weight over 4 weeks were significantly associated with increases in total water intake, and decreases in the half‐life of water in the body, urine potassium, urine osmolality, and BIA reactance. Table 3 describes the estimated mean change in percent body weight associated with a unit change in each of these variables. Each 1 L/day increase in total water intake was associated with a one percent increase in body weight, for example. Multiplying the estimated effect associated with each one mmol/kg change in urine osmolality by 500, a 500 mmol/kg decrease in urine osmolality over 4 weeks was associated with a one percent increase in percent body weight. Change in total water intake explained about one‐third of the within‐person variation in body weight change. Change in urine ADH and plasma aldosterone explained over half of the between‐person variation in body weight change. Change in urine potassium explained one quarter of the within‐person variation and about half of the between‐person variation in body weight change.

**Table 3:** Estimated mean change^a^ in indices of total body water volume associated with unit changes in indices of total water intake and body water flux, regulation, and distribution over 4 weeks of higher water intake

	Indices of total body water volume											
	Body weight change, % of Baseline				Saliva osmolality, mmol/kg				Height‐squared BIA resistance, Hz			
	Change	*P*	R‐squared		Change	*P*	R‐squared		Change	*P*	R‐squared	
	*β * ± SE		W	B	*β * ± SE		W	B	*β * ± SE		W	B
Total water intake												
L/day^b^	1.0 ± 0.3	0.004	0.38	0.00	−13 ± 4	0.005	0.36	0.08	0.7 ± 0.2	0.01	0.37	0.04
mL/kcal intake	0.8 ± 0.3	0.03	0.23	0.00	−8 ± 5	0.1	0.14	0.05	0.2 ± 0.3	0.4	0.05	0.02
mL/kg body weight	0.07 ± 0.02	0.006	0.35	0.00	−0.9 ± 0.3	0.006	0.35	0.05	0.05 ± 0.02	0.01	0.37	0.01
% Change vs Week 2	0.03 ± 0.01	0.008	0.33	0.10	−0.4 ± 0.1	0.02	0.26	0.01	0.02 ± 0.008	0.008	0.40	0.04
Body water flux												
Half‐life of water in the body, % Change^c^	−0.04 ± 0.01	0.002	0.32	0.15	0.2 ± 0.2	0.2	0.06	0.62	0.001 ± 0.01	0.9	0.00	0.27
Half‐life of water in the body, days	−0.2 ± 0.08	0.005	0.28	0.07	1 ± 1	0.3	0.04	0.16	0.02 ± 0.08	0.8	0.00	0.39
Body water regulation												
Thirst, mm^d^	−0.001 ± 0.02	1.0	0.00	0.12	−0.2 ± 0.3	0.6	0.02	0.08	0.02 ± 0.01	0.09	0.16	0.02
Serum osmolality, mmol/kg^e^	−0.06 ± 0.08	0.5	0.02	0.01	2 ± 1	0.1	0.10	0.03	−0.07 ± 0.07	0.3	0.06	0.25
Urine ADH, pmol/L^f^	−0.1 ± 0.03	0.03	0.18	0.52	1.0 ± 0.5	0.046	0.16	0.00	−0.02 ± 0.03	0.50	0.02	0.32
Plasma aldosterone, pg/mL^e^	0.01 ± 0.006	0.08	0.13	0.65	−0.08 ± 0.1	0.4	0.03	0.09	0.01 ± 0.006	0.1	0.15	0.23
Vitamin D 1,25, pg/mL^e^	0.02 ± 0.02	0.2	0.06	0.46	−0.5 ± 0.2	0.04	0.17	0.24	0.004 ± 0.01	0.8	0.01	0.02
Serum Na, mmol/L^e^	0.3 ± 0.2	0.1	0.10	0.27	−0.1 ± 3	1.0	0.00	0.16	−0.1 ± 0.2	0.6	0.01	0.04
Serum K, mmol/L^e^	−1.9 ± 1.2	0.1	0.10	0.15	−16 ± 19	0.4	0.03	0.03	1.1 ± 1.3	0.4	0.04	0.56
RBC K:Na^e^	−0.3 ± 0.2	0.1	0.10	0.02	2 ± 3	0.5	0.02	0.16	−0.2 ± 0.2	0.2	0.08	0.00
Fasting RMR, kcal/day^g^	−0.0009 ± 0.0007	0.3	0.05	0.14	0.03 ± 0.009	0.005	0.29	0.09	−0.001 ± 0.0005	0.02	0.27	0.27
Urine Na, mmol/L^h^	−0.005 ± 0.004	0.3	0.05	0.99	0.1 ± 0.1	0.1	0.09	0.12	−0.002 ± 0.005	0.7	0.00	0.81
Urine K, mmol/L^h^	−0.02 ± 0.007	0.005	0.28	0.46	0.3 ± 0.1	0.02	0.21	0.44	−0.003 ± 0.009	0.7	0.01	0.24
Urine osmolality, mmol/kg^h^	−0.002 ± 0.008	0.02	0.23	0.68	0.03 ± 0.01	0.01	0.24	0.50	−0.002 ± 0.0008	0.8	0.00	0.21
Urine norepinephrine, ug/g crt^f^	−0.007 ± 0.01	0.6	0.01	0.12	0.2 ± 0.2	0.4	0.03	0.19	−0.01 ± 0.01	0.4	0.03	0.64
Systolic blood pressure, mmHg^e^	−0.03 ± 0.03	0.4	0.02	0.04	−0.1 ± 0.5	0.9	0.00	0.45	0.006 ± 0.03	0.8	0.00	0.54
Diastolic blood pressure, mmHg^e^	−0.1 ± 0.06	0.05	0.16	0.06	−0.4 ± 0.8	0.6	0.01	0.13	0.06 ± 0.05	0.2	0.10	0.82
Body water distribution												
BIA height‐squared/reactance, cm^2^/Ω^g^	0.4 ± 0.2	0.01	0.30	0.99	−4 ± 3	0.3	0.06	0.07	0.1 ± 0.2	0.5	0.03	0.87
Manual hematocrit, %^e^	−0.2 ± 0.1	0.1	0.10	0.19	3 ± 2	0.2	0.08	0.02	−0.1 ± 0.1	0.4	0.04	0.77
Automated hematocrit, %	−0.1 ± 0.1	0.3	0.04	0.37	3 ± 2	0.1	0.09	0.00	0.01 ± 0.1	0.9	0.00	0.93
Body water volume												
Saliva osmolality, mmol/kg^d^	−0.02 ± 0.01	0.1	0.09	0.09					−0.03 ± 0.009	0.007	0.32	0.01
BIA height squared/resistance, cm^2^/Ω^g^	0.4 ± 0.3	0.2	0.10	0.88	−12 ± 4	0.007	0.32	0.01				
Body weight, kg^i^	1.5 ± 0.01	<0.001	0.99	0.00	−7 ± 4	0.1	0.09	0.33	0.4 ± 0.3	0.2	0.10	0.35
Body weight, % of Baseline					−4 ± 3	0.1	0.09	0.09	0.3 ± 0.2	0.2	0.10	0.88

a Data presented are estimates from within‐person fixed effects models that include all data from Week 1 to Week 6, *n *=* *5. The *β * ±  SE *β* represent the estimated mean change in each index of total body water (percent body weight change, saliva osmolality, and height‐squared reactance) associated with a unit change in indices of total water intake and body water flux, regulation, and distribution. The W and B represent the within and between R‐squares for each bivariate relationship, respectively.

b The weekly mean intake was estimated from 7 24 h records.

c The half‐life of water in the body was estimated by deuterium elimination rate in Periods 1, 2, and 3.

d Thirst and overnight, water restricted, fasting saliva osmolality was assessed on arrival at the clinic.

e Blood pressure was measured and fasting blood collected 90 min after a 750 mL water bolus.

f Urine ADH and norepinephrine were determined on urine collected after the first morning void until 11 pm on the day before the clinic visit.

g Fasting, recumbent RMR and bioimpedance reactance were determined 60 min after the 750 mL water bolus.

h Urine sodium, potassium, and osmolality were determined on first morning specimen collected at home before the clinic visit.

i Fasting body weight was measured when participants arrived at the clinic.

Decreases in saliva osmolality were significantly associated with increases in total water intake, increases in plasma vitamin D 1, 25, decreases in fasting RMR, urine potassium concentration, and urine osmolality, and increases in height‐adjusted BIA resistance. Change in these variables explained an estimated 36%, 17%, 29%, 21%, 24%, and 32% of the within‐person variation in saliva osmolality, respectively. The magnitude of change was not trivial, considering that the mean total water intake increased by almost 40% relative to baseline; plasma vitamin D 1, 25 increased by over 50% relative to baseline. Fasting RMR changed by over 500 kcal/day for two of the participants (see Table 2).

Increases in height‐squared BIA resistance were associated with increases in total water intake and decreases in fasting RMR and saliva osmolality, explaining about one‐third of the within‐person variation in these variables.

### Associated changes in indices of body water regulation

Table 4 describes the estimated mean change in each index of body water regulation associated with a unit change in other indices of body water regulation between Week 1 and Week 6. Data are not shown for variables that were not associated with other variables. In within‐person change models, on average ± SEM, a one unit decrease in first morning urine osmolality was associated with a 0.005 ± 0.002 mmol/kg (*P* = 0.03) decrease in fasting serum osmolality, a 0.06 ± 0.03 pg/ml (*P* = 0.03) increase in plasma aldosterone, a 0.03 ± 0.01 (*P* = 0.02) increase in plasma Vitamin D 1,25, and decreases in first morning urine sodium of 0.1 ± 0.03 mmol/L (*P* = 0.004) and potassium of 0.1 ± 0.01 mmol/L (*P* < 0.001). Change in urine osmolality explained approximately 20% of the within‐person variation in serum osmolality, plasma aldosterone, and plasma Vitamin D 1,25. Change in urine potassium explained 75% of the within‐person change in urine osmolality. In addition to the relationships described in Table 4, there was a borderline significant (*P* = 0.05) inverse relationship between serum sodium and fasting RMR (−87 ± 42 kcal/day for each mmol/L increase in serum sodium).

**Table 4:**
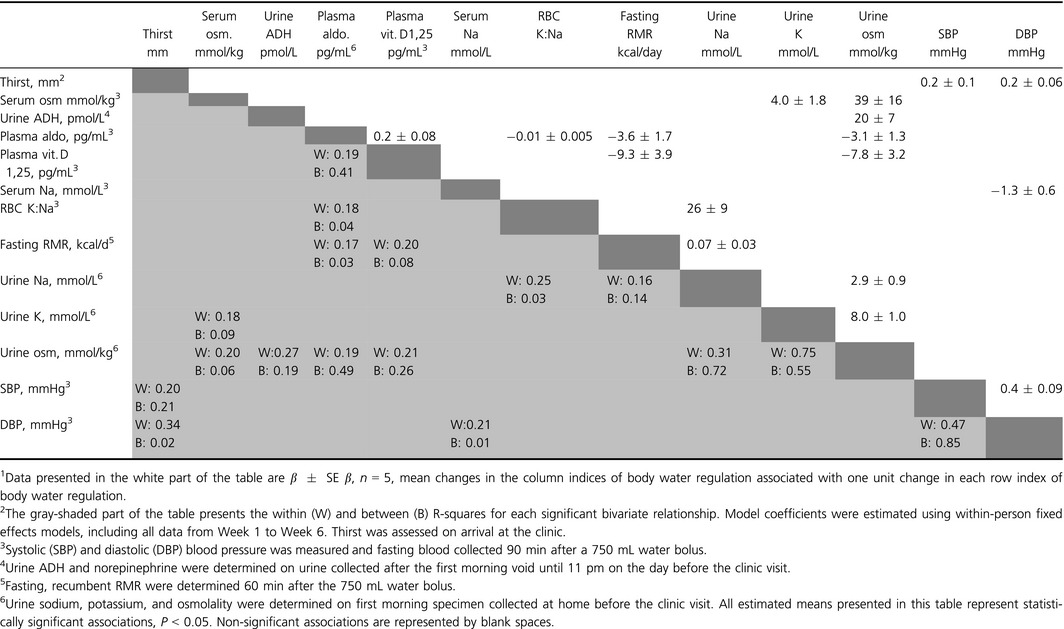
Estimated mean change^a^ in indices of body water regulation associated with a unit change in other indices between Week 1 and Week 6

### Effect modification by baseline status

The magnitude of changes in the half‐life of water in the body, serum sodium, urine potassium, urine norepinephrine, and systolic blood pressure were significantly modified by the level of baseline saliva osmolality. Table 5 reports the mean weekly changes in these indices by level of baseline saliva osmolality. Participants with a saliva osmolality at or above 100 mmol/kg at baseline had significantly smaller decreases in the half‐life of water in the body, greater increases in serum sodium, and greater decreases in urine norepinephrine between Weeks 1 and 6, than participants with saliva osmolality below 100 mmol/kg at baseline.

**Table 5:** Estimated mean^a^ change each week from Week 1 to Week 6 in total water intake and indices of body water flux, regulation, distribution and volume, by baseline saliva osmolality

	Baseline saliva osmolality	
	<100 mmol/kg	≥100 mmol/kg
Total water intake		
L/d^b^	0.3 ± 0.05^j^	0.3 ± 0.06^j^
mL/kcal intake	0.2 ± 0.1^j^	0.2 ± 0.1
mL/kg body weight	4.5 ± 0.8^j^	3.8 ± 0.8^j^
% Change vs Week 2	10 ± 2^j^	8 ± 2^j^
Body water flux		
Half‐life of water in the body, % Change^c^	−9 ± 3	−4 ± 1^j^, ^k^
Half‐life of water in the body, days	−1.4 ± 0.5	−0.5 ± 0.1^j^, ^k^
Body water regulation		
Thirst, mm^d^	−2 ± 2	0.2 ± 1.7
Serum osmolality, mmol/kg^e^	−0.9 ± 0.4^j^	−0.3 ± 0.3
Urine ADH, pmol/L^f^	−1.7 ± 1.0	−1.5 ± 0.5^j^
Plasma aldosterone, pg/mL^e^	7.0 ± 4.6	7.0 ± 3.8
Plasma vitamin D 1, 25, pg/mL^e^	3.0 ± 1.3^j^	2.8 ± 1.9
Serum Na, mmol/L^e^	−0.1 ± 0.2	0.3 ± 0.1^j^, ^k^
Serum K, mmol/L^e^	−0.02 ± 0.03	0 ± 0.02
RBC K:Na^e^	−0.1 ± 0.1	−0.3 ± 0.1^j^
Fasting RMR, kcal/d^g^	7 ± 39	−74 ± 34^j^
Urine Na, mmol/L^h^	−13 ± 9	−10.1 ± 3.8^j^
Urine K, mmol/L^h^	−11 ± 3^j^	−5.3 ± 2.0^j^, ^k^
Urine osmolality, mmol/kg^h^	−99 ± 23^j^	−74.7 ± 19.4^j^
Urine norepinephrine, ug/g crt^f^	3.3 ± 2.8	−3.0 ± 1.5^k^
Systolic blood pressure, mmHg^e^	2 ± 1	−1.1 ± 0.8^k^
Diastolic blood pressure, mmH^e^	−0.1 ± 0.6	−0.7 ± 0.5
Body water distribution		
BIA height‐squared/reactance, cm^2^/Ω^g^		0.3 ± 0.1
Manual hematocrit, %^e^	0 ± 0.2	−0.3 ± 0.2
Automated hematocrit, %^e^	−0.4 ± 0.2	−0.3 ± 0.2
Body water volume		
Saliva osmolality, mmol/kg^d^	−1.3 ± 1.7	−5.3 ± 1.8^j^
BIA height squared/resistance, cm^2^/Ω^g^		0.2 ± 0.1
Body weight, kg^i^	0.2 ± 0.1^j^	0.3 ± 0.1^j^
Body weight, % of Baseline	0.4 ± 0.1^j^	0.4 ± 0.1^j^

a Data are presented as *β * ±  SE *β*, regression coefficients from stratified fixed effect models representing the estimated mean change in outcome each week from Week 1 to Week 6. Results from stratified models for participants with baseline saliva osmolality below 100 mmol/kg, *n *=* *2. Results from stratified models for participants with baseline saliva osmolality below 100 mmol/kg, *n *=* *3.

b The weekly mean intake was estimated from 7 24 h records.

c The half‐life of water in the body was estimated by deuterium elimination rate in Periods 1, 2, and 3.

d Thirst and overnight, water restricted, fasting saliva osmolality was assessed on arrival at the clinic.

e Blood pressure was measured and fasting blood collected 90 min after a 750 mL water bolus.

f Urine ADH and norepinephrine were determined on urine collected after the first morning void until 11 pm on the day before the clinic visit.

g Fasting, recumbent RMR and bioimpedance reactance were determined 60 min after the 750 mL water bolus.

h Urine sodium, potassium, and osmolality were determined on first morning specimen collected at home before the clinic visit.

i Fasting body weight was measured when participants arrived at the clinic.

j Significant change relative to baseline in stratified fixed effect model, *P *<* *0.05.

k Significantly different change over time for participants with baseline saliva osmolality ≥100 mmol/kg versus participants with baseline saliva <100 mmol/kg in mixed model, *P *<* *0.05.

In participants with a saliva osmolality at or above 100 mmol/kg at baseline, increases in percent body weight were associated with increases in height‐squared BIA resistance. Each one percent increase in percent body weight between Week 1 and Week 6 was associated with a mean ± SE increase in height‐squared BIA resistance of 0.7 ± 0.3 Hz (*P* = 0.048). Baseline BIA data were not available for participants with a saliva osmolality below 100 mmol/kg at baseline.

In participants with higher saliva osmolality at baseline, each 1 L/day increase in total water intake, over 4 weeks, was associated with an average 1.2 ± 0.4 percent increase in percent body weight (*P* = 0.013). In participants with saliva osmolality below 100 mmol/kg at baseline, each 1 L/day increase in total water intake was not associated with a significant increase in percent body weight over 4 weeks (0.7 ± 0.4 percent increase in percent body weight, *P* = 0.16).

## Discussion

This study addressed gaps in knowledge about the effects of chronically increased water intake on hydration indices in healthy, young men, under daily life conditions. The study contributes preliminary data regarding longitudinal, long‐term effects of prescribed increases in water intake on indices of body water flux, regulation, distribution, and volume, between‐person variability, and inter‐correlated change in indices. The results are pertinent to debate over the concept of chronic dehydration. Contrary to expectation, the results suggest that chronic total body water deficit may occur in healthy individuals, despite ad libitum access to water.

### Concept of chronic dehydration

Current consensus is that total body water is stable over time in healthy individuals, under daily life conditions, regardless of the volume of water they consume (IOM, 2004; Cheuvront and Kenefick 2016). An increase in water intake is expected to suppress anti‐diuretic hormone, cause urine dilution and excretion, and decrease the half‐life of water in the body in proportion with the increase in water intake. Deuterium elimination is reportedly within 1.3 percent of total water intake (Schoeller and van Santen 1982). Changes in urine excretion are expected to allow serum osmolality and total body water to remain essentially constant over a wide range of water intakes (IOM, 2004).

Contrary to expectation, in this study, indices of body water volume did not remain stable over time. Over 4 weeks, body weight significantly increased and saliva osmolality significantly decreased, despite no significant changes during the study period in environmental conditions, total energy or macronutrient intake, or time spent doing moderate or vigorous physical activity. Urine ADH decreased, and urine dilution occurred, but serum osmolality did not remain stable; it decreased significantly. Between Week 1 and Week 6, the half‐life of water in the body decreased, but not in proportion with the increase in water intake. The results suggest the hypothesis that, for individuals who usually consume less than 2 L/day total water, a sustained increase in drinking water intake of +0.5 L/day might result in slow recovery from an initial <3% total body water deficit.

Increases in body weight and decreases in saliva osmolality were observed with concurrent changes in indices of body water flux and regulation. Plasma aldosterone and vitamin D 1,25 increased. Sodium and potassium excretion decreased. The intracellular RBC K:Na decreased. In within‐person change models, these changes were significantly associated.

A chronic change in total body water is not impossible, given that there are a few plausible mechanisms for this to occur. Decreases in ADH lead to decreased activity of the distal potassium secretory mechanism, which decreases potassium losses under conditions of full hydration and water diuresis (Palmer, 2015). In vitro, and in animals, chronic hypotonicity results in changes in gene expression that favor sodium retention. Chronic hypotonicity stimulates sodium absorption by the kidneys, by increasing expression of aldosterone synthase (LeHoux and Tremblay 1992; Müller 1998), increasing expression of aldosterone receptors (Viengchareun et al. 2009) and ENaC pumps (epithelial sodium channel, aka amiloride sensitive sodium channels) in the distal nephron (Taruno et al. 2008), and recruiting Na‐K pumps (Na‐K‐ATPase) in the collecting duct (Vinciguerra et al. 2004). Chronic hypotonicity increases sodium influx and intracellular sodium concentration in cells from the collecting duct, where the kidney fine tunes total body sodium balance (Niisato and Marunaka 1997, 1999; Vinciguerra et al. 2004; Niisato et al. 2012). In human patients with conditions characterized by chronic hyponatremia, such as chronic renal failure, essential hypertension treated with diuretic medications, and congestive heart failure, RBC, and skeletal muscle cells have a reduced number of Na‐K pumps and increased intracellular sodium concentration (Cheng et al. 1984; Ringel et al. 1987; Toldi et al. 1987; Nørgaard et al. 1990; Baba et al. 1999).

Verbalis and Drutarosky (1988) report results from an experiment involving 193 rats that are essentially the inverse of findings of the present study in healthy young men. Whereas the present study decreased ADH and urine osmolality over 4 weeks, Verbalis and Drutarosky (1988) sustained an increase in urine osmolality over 5 weeks, through infusion of an ADH analog (5 ng/h DDAVP). Whereas, serum sodium increased, RBC K:Na content decreased, and body weight increased in the present study, serum sodium decreased, brain sodium content decreased by 11%, brain potassium content decreased by 17%, and body weight was maintained approximately 5% below starting weight over time (i.e., a chronic total body water deficit) in the rats. DDAVP infusion over 5 weeks produced chronic hyponatremia and hypoosmolality in the rats. The fact that the results in the rats are opposite to the results of the present study, despite significant decreases in plasma osmolality in both studies, generates the hypothesis that sustained decreases in ADH may be a key causal factor for chronic change in electrolyte and total body water balance, whereas hypo‐osmolality, by itself, may not.

Given the descriptive nature of this study, it is not possible to attribute a cause of the change in body weight. In addition to generating hypotheses about change in electrolyte retention, increases in body weight might result from positive energy balance and increases in body fat. The resting, fasting RMR decreased significantly over time for the three study participants with baseline saliva osmolality over 100 mmol/kg. Data were not available to check for concurrent change in fat stores. Equations for estimating FFM and body fat from BIA are not valid under conditions of altered hydration (Mialich et al. 2014).

Data were not available to determine if the observed changes in body weight were associated with increases in total body water. D2O was administered to track elimination rates to index fluid intake and compliance with the Adapt Study protocol. The intent was not to estimate total body water from the D2O, given no expectation of change in this parameter. The D2O doses were not recorded with adequate accuracy to calculate total body water. Further long term studies with gold standard measures of total body water and body fat are needed to check if total body water remains stable in free‐living individuals who consume different amounts of water, as widely believed.

The fact that multiple indices changed together, in concert, suggests that the observed changes in indices of body water regulation and volume were not random or solely attributable to measurement error. Different kinds of specimen were collected at different time points during the protocol (previous day, first morning, and mid‐morning), and analyzed using different instruments and laboratories, by staff blinded to the study protocol and researchers expecting no change in total body water.

Beyond the question of whether or not true increases in total body water occurred for all five study participants, the data raise questions about whether the changes observed in this study represent improvement in hydration or detrimental hyperhydration. Significant, concurrent decreases in fasting serum insulin, homeostasis model assessment (HOMA) index, first morning saliva cortisol (Stookey et al. 2013) and the Tiselius index (data not shown) for the Adapt Study participants suggest that responses to the sustained higher water intake were not detrimental with respect to risk of diabetes, metabolic syndrome, chronic kidney disease, and kidney stones. Among Adapt Study participants with saliva osmolality above 100 mmol/kg at baseline, the higher water intake was associated with a significant decrease in blood pressure. Although the estimated mean change in systolic blood pressure was small (1 mmHg per week), it reflected a decrease in 18 mmHg over 6 weeks for one individual. If larger studies replicate this finding, the small mean weekly change could be clinically relevant for some individuals. The effects of chronic water intake in this study are consistent with short‐term experimental effects of water intake on glycemic control, cortisol, and cardiac vagal control of heart rate and blood pressure (Berneis et al. 1999; Routledge et al. 2002; Perrier et al. 2013). Further studies are needed to determine effects of chronic water intake on chronic disease risk.

### Gaps in knowledge about effects of chronic water intake

#### Effects of prescribed (not ad libitum) water intake

The study participants were instructed to increase intake of drinking water over the baseline amount by 1 L/day for 2 weeks, and then further increase intake to 2 L/day for the following 2 weeks, while maintaining constant their intake of fluids from foods and other beverages. Although participants varied in protocol compliance, all participants maintained an increase in total water intake of at least 0.5 L/day relative to baseline over 4 weeks. Compliance with water intake instruction is notoriously poor for various physiological (e.g., lack of thirst), lifestyle (e.g., habits) and environmental (e.g., lack of available water sources and bathrooms) reasons. To inform the design of future water intake studies, recommendations, or interventions that account for differences between prescribed and actual intake, the effects in this study can be interpreted as effects of a “prescribed sustained increase in water intake of 1–2 L/day” or as effects of an “actual increase in total water intake of more than 0.5 L/day, sustained over 4 weeks.”

Unlike prescribed intake, which can be consciously, deliberately realized, ad libitum water intake is a function of physiological factors, which can be unconsciously suppressed. Oropharyngeal sensors, for example, can cause thirst and drinking to cease before blood osmolality or sodium concentrations decrease in response to fluid intake (Greenleaf 1992). Thirst‐driven fluid intake can be suppressed by electrolyte deficit (Nose et al. 1988a,b) and result in under‐replacement of body fluid over the short‐term (Johnson 1964). Thirst‐driven fluid intake is more sensitive to extracellular dehydration than hypovolemia (Mack and Nadel 1996).

In Week 6, the 7‐day mean water intake for all five participants (range:1.5–2.3 mL/kcal energy intake) was consistent with the Food and Nutrition Board 1989 recommendation of 1.5 mL/kcal of energy expenditure (NRC, 1989). The 7‐day mean total water intake, nevertheless, remained below the 3.7 L/day Adequate Intake set by the IOM in 2004, for four out of five participants. The present results generate the hypothesis that the AI for total water intake of 3.7 L/day, which was designed to prevent acute dehydration, also prevents chronic dehydration. Except for frequent need to urinate and lack of restrooms, the higher water intake did not result in adverse events for the participants.

#### Effects of chronic water intake over the longer‐term

Consistent with previous short‐term studies (Adolph 1947; IOM, 2004), serum osmolality and body weight did not differ significantly from week to week. Consistent with adaptive responses to chronic osmotic challenge requiring lag‐time (Yancey et al. 1982; Lang et al. 1998; Lang 2006), however, significant changes in hydration indices were observed after a delay of several weeks. The results suggest need for long‐term studies of chronic water intake, and indices that cover longer time intervals, such as the 2–3 week half‐life of water in the body, or 120 day life span of the RBC (Piomelli and Seaman 1993).

#### Longitudinal effects of chronic water intake

In this study, with each participant serving as his own control, an average increase in 1 L/day water was associated with significant changes in hydration indices, including change in serum osmolality. These results conflict with findings from cross‐sectional analysis of no difference in serum osmolality between‐people with different levels of water intake (IOM, 2004).

The longitudinal design revealed a surprising increase in serum sodium for participants with baseline saliva osmolality below 100 mmol/kg following sustained higher water intake, contrary to expectation of hemodilution and decreased serum sodium. Although the magnitude of increase was small (ranging from +1 to +3 mmol/L), increases in osmolality of this magnitude are associated with response (Robertson et al., 1976). This result signals potential for U‐shaped or time‐dependent effects of water intake on serum sodium, and need for longitudinal studies of chronic hydration to establish the direction and temporality of effects.

#### Between‐person variability in response to water intake

The five study participants differed in their pattern of response to higher water intake. While serum sodium increased for three participants, it remained stable or decreased for the two others. Observed coefficients of variation were altered by the sustained higher water intake. Whereas urine osmolality had a lower coefficient of variation than saliva osmolality, at baseline, (14% vs. 33%), the reverse was true after 4 weeks of higher water intake (48% vs. 23%).

#### Indices of chronic water intake

In short‐term controlled experiments, serum osmolality is considered to be more sensitive to change in acute total body hydration than saliva osmolality. Cheuvront et al. (2010) report a sensitivity of 80% for saliva osmolality versus 90% and 91% for serum and urine osmolality, respectively, in healthy adults acutely dehydrated by exercise in the heat with fluid restriction. The reported specificity was 83% versus 100% and 91% for saliva versus serum and urine osmolality, respectively. Taylor et al. (2012) report that saliva osmolality correctly classified fewer than 50% of healthy young men who had acutely lost more than 3% body weight by exercise and heat exposure. Ely et al. (2011) report a higher correlation between acute change in TBW and acute change in serum osmolality (*r* = −0.85 to 0.87) than between acute change in TBW and acute change in saliva osmolality (*r* = −0.71 to 0.73).

In the present, longer‐term study, the statistically significant decrease in serum osmolality was small in magnitude (−2 mmol/kg) and within the margin of error for osmometers (±5 mmol/kg). Hydration classification, based on serum osmolality < 295 mmol/kg, did not change over time. Both before and after the increase in water intake, all participants had a serum osmolality below this cutoff. The significant 4‐week changes in indices of body water flux, electrolyte regulation, and total body water, with no significant changes in indices of body water distribution, suggest gradual isotonic total body hydration. Serum osmolality is known to be relatively insensitive to isotonic de/hydration (IOM, 2004).

The results of this study suggest that saliva osmolality may be a more sensitive and specific indicator of change in chronic total body water than serum or urine osmolality. At baseline, only the saliva osmolality classification varied between participants. Only the saliva osmolality classification predicted significantly modified responses to sustained higher water intake over subsequent weeks. Neither serum nor urine osmolality signaled between‐person variability in subsequent changes in sodium retention, RBC K:Na, water turnover, fasting RMR, urine norepinephrine, or blood pressure. Fortes et al. 2015 single out saliva osmolality for use as an indicator of isotonic dehydration. In older individuals, within 30 min of admittance to hospital acute medical care, Fortes et al. 2015 observe that “saliva osmolality was able to detect water‐and‐solute‐loss dehydration, for which a measurement of plasma osmolality would have no diagnostic utility.” Consistent with saliva osmolality as an indicator of isotonic dehydration, saliva osmolality increases significantly more (by 26 vs. 6 mmol/kg) when 1% body water is lost due to cycling at moderate intensity in the heat than when 1% body water is lost passively from sitting in the heat (Muñoz et al. 2013).

Saliva secretion is under autonomic regulation (Proctor and Carpenter 2014). Aldosterone regulates saliva osmolality (Lauler et al. 1962; Harboway 1976; Riad et al. 1986). As plasma aldosterone increases, saliva sodium and osmolality decrease. In so far as saliva osmolality reflects delayed effects of genomic upregulation of aldosterone and/or Na‐K‐2Cl transport (Kidokoro et al. 2014), saliva osmolality could be a plausible index of chronic hydration.

After 4 weeks of sustained increased water intake, the agreement between serum, urine and saliva classifications improved such that five out of five participants were classified as hydrated based on serum<295 mmol/kg and urine osmolality<800 mmol/kg, and four out of five participants were classified as hydrated based on saliva osmolality<100 mmol/kg. Agreement between these three indices depends on the magnitude of change in total body water (Popowski et al. 2001; Smith et al. 2012). Cheuvront et al. (2010) suggest that agreement between indices is a function of population heterogeneity in homeostatic control. It is unknown if population heterogeneity in homeostatic control is a function of between‐person differences in chronic hydration.

Acknowledging that there is no one gold standard measure of hydration (Ritz 1998; Shireffs 2003; Armstrong 2007), and given potential for chronic total body water deficit to reflect isotonic dehydration, future studies of chronic hydration should include indices besides serum osmolality.

#### Interdependent effects

In this study, the pattern of change in indices was significantly modified by baseline saliva osmolality. Among study participants who had a saliva osmolality above 100 mmol/kg at baseline, increases in body weight were significantly associated with increases in serum sodium, decreases in the RBC K:Na, and smaller decreases in the half‐life of water in the body, suggesting that some of the increase in water was retained, not excreted. The significant decreases in RBC K:Na were observed with decreases in whole body resting energy expenditure, and urine norepinephrine.

In contrast, participants with lower baseline saliva osmolality did not increase sodium retention and had a significantly greater decrease in the half‐life of water in the body, almost equal in magnitude (−9 ± 3%) to the percent increase in water intake (10 ± 2%). Further studies are needed to describe changes in saliva osmolality and sodium retention in response to chronic change in water intake.

Interdependent metabolic and physiologic mechanisms promise complexity for future studies in terms of confounding and/or effect modification. The renin‐angiotensin‐aldosterone system (RAAS) interacts, for example, with calcium regulatory systems (Vaidya et al. 2015). Chronic aldosterone stimulates parathyroid hormone in healthy humans (Brown et al. 2014, 2015). Higher vitamin D downregulates renin synthesis and decreases RAAS activity, independent of Ca (de Martin 2011). Higher vitamin D 1,25 and serum calcium downregulate Na‐K pump activity in smooth muscle and RBCs, resulting in increased intracellular sodium (Brickman et al. 1986).

The present results generate hypotheses such as the metabolic and physiologic effects of acute dehydration vary by baseline chronic hydration state; the long‐term health effects of urine osmolality depend on level of saliva osmolality.

### Limitations

This study has limitations as a secondary analysis of data from a small clinical study involving only five participants. The small sample size and selection criteria limit generalizability. The study was originally designed to focus on cell hydration, not total body hydration.

Various sources of measurement and confounding error limit interpretation of the present results. Although no significant systematic differences in solute intake were observed, diet records are vulnerable to measurement error, particularly for micronutrient intakes. Unobserved heterogeneity in sodium intake cannot be ruled out, as standardized meals were not provided in this study.

As mentioned above, increases in body weight may reflect increases in body fat as opposed to body water. The D2O dose might not have been fully equilibrated at the time of the blood draw, underestimating the background deuterium enrichment, despite reports that 90 min is adequate time for equilibration (Jankowski et al. 2004). If total body water did increase over time, the equation for estimating water turnover can only be interpreted as the turnover rate of the isotope, not body water per se (Nagy and Costa 1980).

Stress‐induced cortisol can bind the Na‐K pump and act like aldosterone. Decreases in the RBC K:Na can reflect RBC age, to the extent that RBC lose pumps as they mature. Cortisol and older RBC likely do not explain the present results, however, because cortisol was lower in Week 6 and the number and proportion of reticulocytes was significantly increased in Week 6 (Stookey et al. 2013). Nevertheless, given potential for error, further studies are needed to confirm the present findings.

## Conclusions

This study reported preliminary data regarding the concept of chronic dehydration, long‐term within‐person effects of a sustained increase in water intake on hydration indices, and potential for effect modification in chronic hydration studies by baseline status and interdependent regulatory mechanisms. The analysis generated hypotheses regarding effects of sustained higher water intake on increases total body water, gradual isotonic retention of potassium and/or sodium, and saliva osmolality as a sensitive and specific index of chronic hydration. Further research on water intake and chronic de/hydration is warranted.

## Conflict of Interest

JS received unrestricted funds from Danone Research for the original research study, and is an occasional consultant for Danone Research. The other authors have no conflicts to disclose.
